# Current Surgical Approaches to Managing Infective Endocarditis With Aortic Root Abscess

**DOI:** 10.31083/RCM47800

**Published:** 2026-08-20

**Authors:** Lanxin Ye, Fanyu Chen, Weiteng Wang, Oudi Chen, Hongkun Qing, Lixi Gan, Xuhua Jian

**Affiliations:** ^1^Guangdong Cardiovascular Institute, Guangdong Provincial People’s Hospital, Guangdong Academy of Medical Sciences, 510060 Guangzhou, Guangdong, China; ^2^Department of Cardiovascular Surgery, Guangdong Provincial People’s Hospital, Guangdong Academy of Medical Sciences, Southern Medical University, 510060 Guangzhou, Guangdong, China; ^3^State Key Laboratory of Cardiovascular Disease, Fuwai Hospital, Chinese Academy of Medical Science, Peking Union Medical College, 100037 Beijing, China; ^4^Department of Echocardiography, Guangdong Provincial People’s Hospital, Guangdong Academy of Medical Sciences, Southern Medical University, 510060 Guangzhou, Guangdong, China

**Keywords:** infective endocarditis, aortic root abscess, current advancements

## Abstract

Aortic root abscess (ARA) is a life-threatening complication of infective endocarditis (IE) associated with high mortality despite advances in diagnosis and treatment. We conducted a focused review of the clinical characteristics, diagnosis, imaging modalities, causative pathogens, treatment strategies, and outcomes of ARA. This review summarizes results from studies evaluating different surgical approaches for ARA and discusses emerging diagnostic and therapeutic innovations. The mortality rate of ARA complicating IE ranges from 12.2% to 30%. Early diagnosis remains challenging, and multiple surgical approaches are available. The choice of surgical approach depends primarily on the extent and anatomical distribution of the abscess. This review underscores the need for improved diagnostic strategies and optimized surgical strategies to enhance outcomes and long-term prognosis in patients with ARA.

## 1. Introduction

Abscess formation is defined as the presence of necrotic tissue in the aortic root [1], aortic annulus, and aortic–ventricular discontinuity in the setting of infection, or as the presence of purulent material infiltrating necrotic areas involving the valve annulus or adjacent myocardial structures [2]. Epidemiological studies show a rising incidence of infective endocarditis (IE), with rates ranging from 5 to 14.3 cases per 100,000 person-years [3]. Aortic root abscess (ARA), a severe complication of IE, carries a relatively high mortality rate, reported between 12.2% and 30% [4].

ARA develops in 10% to 40% of patients with aortic valve endocarditis [5] and in 10% to 37% of patients with prior aortic root surgery or valve replacement [6].

In recent years, the sensitivity of the Duke IE Criteria has declined in individuals older than 65 years. This decline is attributed to the increasing prevalence of IE in older adults and the high prevalence of comorbidities. The presence of ARA further increases diagnostic complexity and mortality. Given these challenges, management often requires a multidisciplinary endocarditis team, including infectious disease physicians, microbiologists, cardiologists, imaging specialists, and cardiac surgeons, who collaboratively evaluate patients and assist perioperative management [7].

**Fig. 1.**
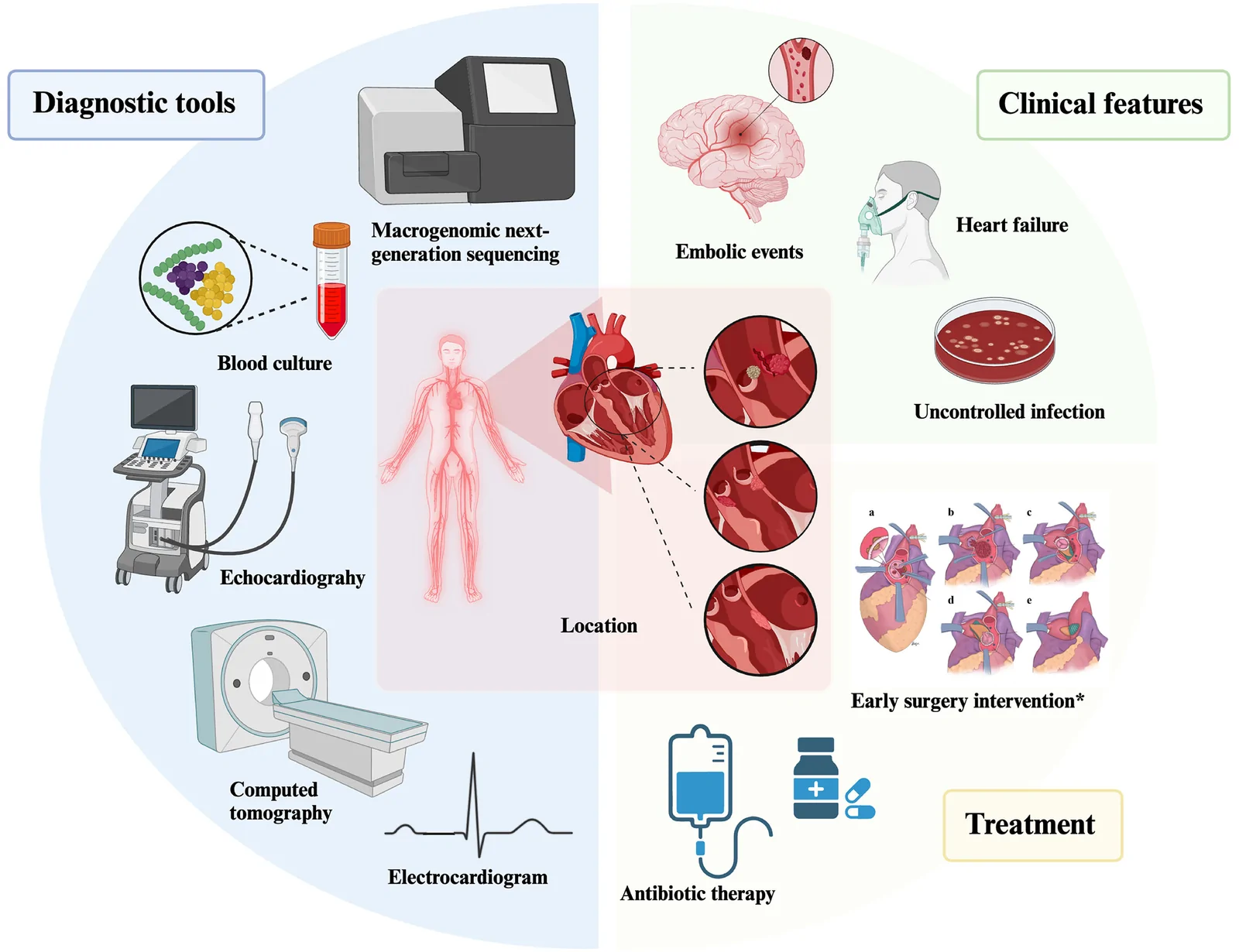
**Contemporary surgical management of aortic root abscess**. *Reprinted from “Application of Commando and hemi-Commando procedures in the reconstruction of intervalvular fibrous body” by Wang et al. [8], published in the Chinese Journal of Clinical Thoracic and Cardiovascular Surgery (2024), with permission from West China Medical Publishers and the original authors.

This article reviews the epidemiology, etiology, clinical presentation, diagnosis, causative microorganisms, imaging, treatment, and outcomes of ARA associated with IE (Fig. 1, Ref. [8]).

## 2. Methods

This study was conducted as a narrative review with a structured literature search. A comprehensive search of PubMed, Embase, and Web of Science was performed to identify relevant studies published between 1990 and 2025. The following keywords were used in various combinations: “infective endocarditis”, “periannular abscess”, “aortic root abscess”, “aortic root replacement”, “patch repair”, and “aortic valve replacement”.

Inclusion criteria were as follows: (1) studies reporting on the diagnosis or surgical management of infective endocarditis with aortic root abscess; (2) studies providing clinical outcomes or surgical details; (3) randomized controlled trials, registry-based studies, retrospective cohort or series analyses, selected review articles, or clinical guidelines.

Exclusion criteria included: (1) case reports with insufficient clinical data; (2) conference abstracts; (3) non-English publications; and (4) studies not directly related to periannular involvement.

Study selection was performed through title and abstract screening followed by full-text review. A Preferred Reporting Items for Systematic Reviews and Meta-Analyses (PRISMA)-style flow diagram summarizing the screening and inclusion process has been provided.

Given the narrative nature of this review, a formal risk-of-bias assessment was not performed systematically; however, greater emphasis was placed on higher-quality studies, including multicenter analyses and those reporting long-term surgical outcomes.

## 3. Epidemiology and Pathology

In a large-scale study of IE, 10% of patients had echocardiographic evidence of abscess, with ARA being the most common form [9]. A meta-analysis of six studies found that approximately 26% of IE cases had root abscesses [4], and 46% of aortic valve IE cases had ARA [10].

Multiple studies have shown that ARA is more common in prosthetic valve endocarditis (PVE) [11] (Ramos-Martínez et al. [12], 34% vs. 13.2%; *p* < 0.0001; Weber et al. [13], 51.9% vs. 26.6%; *p* = 0.001; EuroENDO registry, 19% vs. 8%; *p* < 0.0001 [9]), with mortality rates reaching 50% in PVE [14]. The presence of abscesses is an independent predictor of in-hospital mortality [9], a high-risk factor for IE recurrence [7], and is associated with more frequent cardiac surgery. Research has also found that perivalvular extension is the most common cause of uncontrolled infection after transcatheter aortic valve replacement (TAVR) among patients with IE, with approximately 80% presenting as periannular abscesses [15].

IE is primarily caused by *streptococci* and *staphylococci* (Table 1, Ref. [1,16,17,18,19,20,21,22,23,24]), with *staphylococci* known for biofilm formation and toxin production, which lead to persistent infection and tissue damage. Infection can spread from the interventricular septum to the mitral valve, especially after antimicrobial therapy.

**Table 1.** **Microorganisms in infective endocarditis with aortic root abscess: summary of key studies**.

Author	Year	*Staphylococci*, n (%)	*Staphylococcus aureus*, n (%)	Other *Staphylococcus* spp., n (%)	*Streptococcus* spp., n (%)	*Enterococci*, n (%)	Culture negative, n (%)	Total, n
Elderia et al. [21]	2024	56 (40.3)	33 (23.7%)	23 (16.5%)	46 (33.1)			139
Tomšič et al. [23]	2023	20 (34)	10 (17)	10 (17)	17 (28)	6 (10)	10 (17)	60
Li et al. [20]	2023	31 (39.2)	22 (27.8)	9 (11.3)	6 (7.5)	5 (6)	24 (30)	79
Harris et al. [16]	2022	9 (18)			19 (38)	1 (2)	13 (26)	50
Yang et al. [18]	2021	59 (33)	27 (15.1)	32 (17.9)	53 (29.6)	25 (14)	32 (17.9)	179
Leontyev et al. [22]	2016	40 (27)	12 (8)	28 (19)	22 (15)	13 (9)	63 (42)	150
Kirali et al. [24]	2016	10 (37)	8 (29.6)	2 (7.4)	4 (14.8)	3 (11.1)	9 (33.3)	27
Lee et al. [17]	2014	6 (12.3)	4 (8.2)	2 (4.1)	21 (42.9)	3 (6.1)		49
Leontyev et al. [1]	2012	49 (40.2)	31 (25.4)	18 (14.7)	28 (22.9)	18 (14.7)	21 (17.2)	122
David et al. [19]	2007	62 (53.0)	40 (34.2)	22 (18.8)	19 (16.2)	5 (4.3)	5 (4.3)	117

## 4. Diagnosis

Diagnosis of ARA relies on microbiological and imaging methods, and early detection improves prognosis in ARA patients.

### 4.1 Clinical Features

Patients with ARA usually present with persistent fever and a heart murmur. Patients also have congestive heart failure with Osler nodes, Roth spots, and Janeway lesions. Some patients develop embolic complications and cardiac conduction abnormalities [7]. If untreated, ARA can cause severe valvular dysfunction, prosthetic valve fissure, heart block, pseudoaneurysm, coronary flow obstruction, and fistulae. Poorly controlled heart failure is closely linked to ARA [7].

Atrioventricular block (AVB) is present in approximately 25% of patients with ARA and is more common in the aortic annulus than in the mitral annulus. A high degree of AVB is observed in about 10% of ARA cases [25]. Moreover, ARA is associated with a greater prolongation of the electrocardiographic PR interval compared with aortic valve endocarditis without abscess [26].

### 4.2 Microbiological Diagnosis

#### 4.2.1 Blood and Valve Culture

Positive blood culture results are the cornerstone of ARA diagnosis, as specified in the 2023 Duke-ISCVID criteria. Blood cultures can identify pathogenic microorganisms, but 2%–40% of IE cases are culture-negative, with one study reporting a rate of 71% [27].

In addition to blood cultures, tissue cultures can be used to identify pathogenic microorganisms in ARA. However, tissue samples are only collected during surgery; therefore, they cannot be used for preoperative confirmation of diagnosis or guiding the formulation of preoperative antibiotic regimens. Tissue culture also has a high false-negative rate and a high risk of contamination during sample handling. Studies have shown that blood and valve cultures have sensitivities of 29.3% and 16.2%, respectively [28]. Culture-combined macrogenomic next-generation sequencing (mNGS) has a sensitivity of up to 89.9%, a specificity of 72.7%, and an area under the curve (AUC) of 0.813 (*p* = 0.001) [28].

Excised valve tissue should be evaluated microbiologically and histopathologically, especially in cases of preoperative diagnostic uncertainty or suspected mixed infections. Given the possibility of mixed infections, additional microbiologic testing remains necessary even if a microbiological diagnosis has been established preoperatively [29].

#### 4.2.2 Macrogenomic Next-Generation Sequencing

mNGS is a technique that uses next-generation sequencing to detect microbial DNA in clinical samples [30,31]. Many studies have reported excellent sensitivity and specificity for mNGS [28,32]. Indeed, mNGS has shown good diagnostic performance across different specimen types. Tissue-based mNGS achieved the highest accuracy (83%) with an AUC of 0.74 (sensitivity 86%, specificity 63%). Arterial and venous blood mNGS had lower accuracies (54% and 55%) with AUCs of 0.63 and 0.68, respectively. After multidisciplinary diagnostic evaluation and clinical reassessment, the overall diagnostic performance of mNGS improved significantly, with accuracy reaching 86%, and the AUC increasing to 0.92 (*p* < 0.001). In blood culture-negative endocarditis (BCNE), valve tissue mNGS markedly outperformed conventional tissue culture. mNGS yielded an accuracy of 88% and an AUC of 0.88 (sensitivity 76%, specificity 100%), whereas tissue culture showed only 22% accuracy and an AUC of 0.53 [32].

Current guidelines from the American Association for Thoracic Surgery (AATS) recommend PCR testing of resected valve tissue to identify causative pathogens [33]. Compared with conventional culture methods, next-generation sequencing enables the detection of copathogens and low-abundance organisms that are otherwise difficult to identify [30]. Emerging evidence shows that mNGS improves pathogen detection in BCNE. The main advantages of mNGS include a short turnaround time, with results typically available within 24–48 hours of test initiation [34]. Moreover, mNGS results support pathogen-directed escalation or de-escalation of antimicrobial therapy, reducing unnecessary broad-spectrum antibiotic use in patients with BCNE [32]. However, the high cost of mNGS remains a significant disadvantage [34].

### 4.3 Imaging Techniques

#### 4.3.1 Echocardiography

Echocardiography is crucial for diagnosing ARA, assessing cardiac function, and predicting patient prognosis. Abscesses may appear as thickened, inhomogeneous, densely echogenic, or echogenic hyaline [35]. Early abscesses may present only as nonspecific perivalvular thickening for several days and are more readily recognized when they gradually evolve into a cavity.

Echocardiography has a sensitivity of 63% and a specificity of 90% for detecting abscesses [36], with transesophageal echocardiography (TEE) showing higher sensitivity (up to 86%) and specificity (91%) [6]. TEE provides an improved assessment of PVE and perivalvular abscesses and more comprehensively depicts the cardiac and vascular structures affected by infection, which is crucial for surgical planning [37,38]. However, TEE has several limitations, including suboptimal sensitivity due to relatively low spatial resolution and prosthetic valve-related artifacts that may require further evaluation [39].

Transthoracic echocardiography (TTE) is routinely performed, but the diagnostic accuracy of the technique decreases to 48% in the presence of prosthetic valves, difficult-to-visualize anatomical locations, calcifications, and electrophysiologic devices [37,40]. For patients with a positive initial TTE and cardiac complications, early TEE is recommended. Repeat examinations are advised when abscess or fistula formation becomes more apparent [7]. When TEE is unavailable or delayed, early TTE can still identify high-risk patients and guide diagnosis in some cases (Table 2) [41].

**Table 2.** **Summary of the imaging methods for infective endocarditis**.

Modality	Principle	Major diagnostic targets	Diagnostic performance	Strengths	Limitations	Clinical indications
Transthoracic echocardiography	Transthoracic ultrasound imaging	Vegetations; valvular dysfunction; cardiac hemodynamics	Moderate sensitivity; high specificity	Widely available; noninvasive; first-line examination	Limited sensitivity for abscesses and prosthetic valves; operator-dependent	Initial screening in suspected IE
Transesophageal echocardiography	Transesophageal ultrasound with high spatial resolution	Vegetations; perivalvular abscesses; prosthetic valve involvement; fistulae	High sensitivity and specificity	Superior visualization of periannular structures; essential for surgical planning; superior to TTE for PVE and CIED leads	Semi-invasive; may miss very early abscess formation	Suspected IE with negative or inconclusive TTE; evaluation of complications
Cardiac computed tomography	Contrast-enhanced anatomical imaging	Perivalvular abscesses; pseudoaneurysms; fistulae; prosthetic valve anatomy	High sensitivity for perivalvular complications	Excellent spatial resolution; complementary to echocardiography in PVE	Radiation exposure; contrast-related risks; may miss small, mobile vegetations; no hemodynamic assessment	Assessment of perivalvular extension; preoperative evaluation
Positron emission tomography/computed tomography	Metabolic imaging using [18F]-FDG uptake	Active infection; prosthetic valve endocarditis; extracardiac septic emboli	High sensitivity, especially in prosthetic valves	Detects metabolically active infection; useful in BCNE	False positives due to inflammation; limited availability	BCNE; suspected PVE; CIED infection
SPECT/CT	Radiolabeled leukocyte imaging	Active inflammatory and infectious lesions	Moderate sensitivity; high specificity	High specificity for infection	Lower spatial resolution; time-consuming preparation	Infection in complex PVE

TTE, transthoracic echocardiography; CT, computed tomography; IE, infective endocarditis; PVE, prosthetic valve endocarditis; CIED, cardiovascular implantable electronic device; BCNE, blood culture-negative endocarditis; SPECT, single-photon emission computed tomography; [18F]-FDG, 18F-fluorodeoxyglucose.

#### 4.3.2 Computed Tomography

Computed tomography (CT) can reveal ARA as fluid collections surrounded by enhancing inflammatory tissue [42]. Cardiac CT with or without contrast can detect root abscesses, especially when echocardiography results are negative [43]. Owing to the higher spatial resolution associated with CT, this approach has greater sensitivity than TTE and TEE for detecting abscesses and other complications (81% vs. 36% and 81% vs. 45%, respectively). The combination of CT and echocardiography increases the sensitivity of abscess detection to 100% [36].

Studies have shown that electrocardiography (ECG)-gated CT is more sensitive than TEE for identifying ARA, abscess extension, fistula formation, and valve rupture, and is an effective tool for visualizing abscesses [6,44].

#### 4.3.3 Nuclear Imaging: Positron Emission Tomography/CT (PET/CT) and Single-Photon Emission Computed Tomography/CT (SPECT/CT)

Detection of abnormal activity around valves or prostheses by [18F]-FDG PET/CT or white blood cell (WBC) SPECT/CT is considered a major imaging criterion for the diagnosis of IE in the 2023 ESC guidelines [7]. The sensitivity of WBC SPECT/CT has been reported to range from 64% to 90%, and specificity from 36% to 100%. Diagnostic accuracy can be significantly enhanced when periprosthetic abscesses are considered [7].

However, WBC SPECT/CT requires extensive blood processing to prepare the radiopharmaceutical, has a long acquisition time, and has lower spatial resolution and photon-detection efficiency than [18F]-FDG PET/CT [45]. When ARA is suspected but not demonstrated on echocardiography, PET/CT can be used to detect abnormal perivalvular activity, and [18F]-FDG PET/CT is superior to echocardiography and CT for detecting early abscesses [40,46].

#### 4.3.4 Electrocardiogram (ECG)

Although not a first-line imaging test, ECG is valuable for the early detection of ARA. Disruption of the aortic valve annulus in ARA anatomically increases the risk of AVB and pacemaker dependence, which is characterized by prolonged PR intervals and varying degrees of atrioventricular block, depending on the extent of infection spread [26,47].

Many studies have shown that AVB is a predictor of perivalvular complications. The positive predictive value of new-onset AVB for abscess formation is 88% [48]. However, in some cases, ARA may also present with chest pain and ischemic ECG changes [48], due to occlusion of the left anterior descending artery and circumflex arteries from extrinsic compression by the abscess, or due to valvular failure, leading to global subendocardial ischemia [49].

## 5. Treatment

### 5.1 Antibiotic Therapy

Antibiotic therapy can control ARA by eradicating local infection, preventing its spread, and reducing embolic events. Research indicates a significant reduction in embolic risk within the first two to three weeks of effective antibiotic therapy [50,51]. Since infected heart valves confer bacterial tolerance, antibiotics need to be administered for a sufficiently long duration [52], especially in PVE (>6 weeks), which should be treated longer than native valve endocarditis (NVE) (2–6 weeks) and typically still requires about 2–6 weeks of antibiotic therapy after surgery [7].

In long-term antibiotic therapy for ARA, antibiotic selection and duration depend on the type of IE. For NVE and late PVE, regimens should cover *staphylococci*, *streptococci*, and *enterococci*. In cases of early PVE or healthcare-associated IE, treatment regimens should also include coverage for methicillin-resistant *Staphylococcus aureus* and *enterococci*. In patients with Methicillin-Sensitive Staphylococcus aureus (MSSA) IE and ARA, nafcillin is recommended for at least 6 weeks [53]. Third-generation cephalosporins and quinolones are usually recommended for infections caused by the HACEK group, as some of these bacteria produce β-lactamases [53].

A small proportion of patients with perivalvular infections can be managed nonoperatively, including those with small abscesses (<1 cm), no heart block, no echocardiographic evidence of abscess progression during treatment, and no valve dehiscence or significant insufficiency. Continuous TEE monitoring should be performed in these patients during and after antimicrobial therapy. Furthermore, in hemodynamically unstable patients with a poor prognosis, or in those with neurological complications, who are not candidates for surgery, prolonged antibiotic therapy is required [7,12]. The main limitation of antibiotic therapy is bacterial resistance; some bacteria may persist and continue to grow after treatment discontinuation, necessitating surgical intervention.

### 5.2 Surgical Intervention

For most patients, antibiotic therapy alone does not completely eradicate infection. The high risk and complications associated with ARA frequently necessitate surgical intervention.

Surgeons are particularly concerned about surgical management of ARA, due to higher early mortality, increased risk of infection recurrence, and lower long-term survival rates. In a study by Leontyev et al. [1], the perioperative mortality rate was 25%, 15 patients required reoperation for recurrent IE, and the 1- and 5-year survival rates were 55 ± 4% and 50 ± 4%, respectively. In the Bristol Institute study [16], in-hospital mortality was 12%, and 27% of patients required reoperation. Sak Lee and colleagues [17] reported a perioperative mortality rate of 12%, with a follow-up period of 68.7 ± 40.4 months and no recurrence of infection. Yang et al. [18] reported a surgical mortality rate of 8.4% and a 10-year survival rate of 40%. A meta-analysis of seven studies reported a 30-day mortality of 20% and an overall 1-year reoperation rate of 5.2% [4].

Most studies agree that ARA increases the risk of surgical mortality and subsequent complications. Data from the EURO-ENDO registry suggested that the presence of abscesses is an independent predictor of in-hospital mortality in PVE patients (odds ratio (OR) 1.78; 95% confidence interval (CI) 1.3–2.44) [9]. However, some studies found no significant differences in operative mortality rates (8.4% vs. 3.8%; *p* = 0.11) or long-term survival rates (41% vs. 43%; *p* = 0.36) between patients with ARA and without ARA [18], indicating that, when complete debridement is achieved, the presence of abscesses is not necessarily an independent risk factor for operative mortality. Many authors believe that, despite the increased complexity of abscess surgery, the key determinant of outcome is thorough debridement of the abscess and infected tissues; the type of aortic root graft appears to have less impact on surgical outcomes or long-term survival [18,19,54,55]. Nevertheless, ARA remains a risk factor for all-cause mortality and late reoperations and still requires timely surgical intervention, as highlighted in the 2023 ESC guidelines [7].

The surgical approach for ARA depends on the extent of infection. Common surgical options for treating ARA include aortic valve replacement (AVR), aortic root replacement (ARR), and patch repair/reconstruction (PR), with AVR and ARR being the most widely used techniques.

#### 5.2.1 Timing of Surgery and Risk Factors

Many studies support early surgery in patients with ARA, and even in those with destructive penetrating lesions [56], especially in PVE, as it can be life-saving for patients with aortic valve prostheses infected within 1 year of valve implantation [57]. One study found that early valve replacement benefited a subgroup of patients with abscesses [58], and another demonstrated that early surgery improved early survival [59], with no significant difference in mortality between emergency surgery and elective surgery after antibiotic therapy.

In the presence of systemic arterial pressure, abscesses can lead to perforation and formation of fistulous tracts, resulting in intracardiac or pericardial shunts, a complication associated with a mortality rate as high as 41% [60]. Infection can extend to the aortic–mitral valve continuity or directly involve the mitral valve, thereby increasing surgical risk. 

Multivalvular involvement [16], PVE, liver dysfunction, renal failure requiring dialysis, and postoperative sepsis [18] are major risk factors for perioperative mortality.

#### 5.2.2 Thorough Debridement and Aortic Valve Replacement

The abscess most commonly affects the aortic valve annulus, followed by the subvalvular fibrous triangle, aortic-mitral continuity, and, in some cases, the mitral valve (Table 3, Ref [1,17,19,24,61]). When an abscess involves the aortic valve, it often occurs at its weakest point, the membranous septum and the atrioventricular junction [62]. This anatomical vulnerability may lead to potential postoperative complications such as AVB. During surgery, meticulous attention to the anatomy is essential for adequate exposure and safe debridement. Infection, myocardial edema, and suture tension can all contribute to AVB, often requiring temporary pacemaker placement. Some patients may revert to a normal rhythm after resolution of inflammation, whereas others may require medication or a permanent pacemaker implantation for persistent high-grade block.

**Table 3.** **Summary of studies on the location of abscesses**.

Author	Year	Aortic annulus, n (%)	Intervalvular fibrous body, n (%)	Mitral, n (%)	Interventricular septum, n (%)	Aortic atrial discontinuity, n (%)	Aortic–ventricular discontinuity, n (%)	Total, n
Kirali et al. [24]	2016	10 (37)	6 (22.2)		1 (3.7)	3 (11.1)	7 (25.9)	27
Lee et al. [17]	2014	11 (22)	16 (33)	4 (8)		3 (6.1)		49
Leontyev et al. [1]	2012	90 (52.3)	81 (47.1)	21 (12.2)		16 (9.3)	7 (4.1)	172
David et al. [19]	2007	73 (54)	42 (31)	22 (16)	22 (16)			135
Yankah et al. [61]	2005	62 (38.5)		36 (22.4)	8 (5)	11 (6.8)	83 (51.5)	161

Complete debridement of the abscess cavity is essential. Closure options include direct suturing or patch repair with autologous or bovine pericardium. In chronic abscesses, cavities may be left open after thorough disinfection, sometimes with povidone–iodine irrigation [2]. However, patch repair is not recommended according to the 2023 ESC guidelines, as this approach may increase the risk of abscess recurrence, perivalvular leak, and pseudoaneurysm; in some cases, leaving the cavity open after radical debridement is preferred [7].

When infection is limited to isolated sinuses, AVR combined with PR is recommended. Li et al. [20] advocated using a robust bovine pericardial patch for larger abscesses and aorto–ventricular discontinuity. PR yields acceptable outcomes, with an in-hospital mortality of 11.3% and overall survival rates of 79%, 69%, and 65% at 1, 5, and 7 years, respectively [20]. One patient experienced recurrent infection. The survival rate for NVE was slightly higher than for PVE, not only in single-valve endocarditis (81% vs. 80%; *p* = 0.589) but also in multivalve endocarditis (82% vs. 61%; *p* = 0.132). However, this single-center study included only 79 patients and lacked a sufficient sample size to stratify outcomes by the type and extent of local pathology [20]. Generally, most patients undergoing PR had smaller abscesses and required less extensive surgical reconstruction. Specifically, in cases with localized, well-demarcated abscess cavities, preserved annular integrity, hemodynamic stability, and no evidence of extensive root destruction, meticulous debridement followed by patch reconstruction can yield satisfactory outcomes.

Long-term follow-up and meta-analyses of clinical trials indicate improved long-term survival among patients receiving mechanical valves (log-rank test, *p* = 0.03) [63]. However, these patients have longer hospital stays and higher in-hospital complication rates (55.9% vs. 48.6%); paradoxically, the in-hospital mortality rate is slightly lower (4.4% vs. 4.9%) [64]. Currently, there is no convincing evidence of a difference in the risk of early or late endocarditis between mechanical and bioprosthetic valves.

A study comparing AVR, AVR combined with PR, and ARR found no significant differences in 30-day and 1-year mortality rates; however, the AVR plus PR group had a higher rate of infection recurrence than the ARR group. Since patients selected for isolated AVR generally have less severe disease, AVR plus PR and ARR are usually preferred in more extensive cases. ARR appears more effective for eradicating infection and reducing recurrence rates in patients with extensive infection. PR can reduce the need for full root replacement; however, the final surgical choice still depends on the experience-based assessment of the surgeon.

Multiple studies suggest that patients undergoing AVR instead of ARR may face a higher risk of reoperation due to recurrent infections or non-structural valve deterioration. During surgery, it is sometimes necessary to remove the entire annulus and at least 3–5 mm of surrounding tissue to ensure thorough debridement, which leads many surgeons to favor ARR in most cases [4,16,21,22].

#### 5.2.3 Aortic Root Replacement

For extensive infections involving the entire root or abscesses affecting more than one-third of the aortic annulus, ARR is often preferred [22]. Compared with AVR and PR, ARR requires longer operative and cardiopulmonary bypass times, which may increase in-hospital mortality. However, multiple studies show lower recurrence and reoperation rates with ARR, reflecting the more radical debridement of infected tissue [16,21]. This was confirmed in a study by William M. Harris et al. [16], in which ARR was associated with a 16% increase in late mortality but a 17% decrease in reoperation rates compared with PR. Similarly, in the study by Elderia et al. [21], Bentall surgery, compared with isolated AVR or AVR plus PR, had a lower recurrence rate (0% vs. 26.7%; *p* = 0.095). A meta-analysis by Chen et al. [4] also showed lower recurrence rates and a 50% reduction in the risk of reoperation with ARR.

Several retrospective series have reported lower reinfection rates and improved long-term survival in patients undergoing ARR when extensive annular or root involvement is present. These findings support the concept that radical surgical eradication of infected tissue is critical in complex IE. ARR should be strongly considered in patients with circumferential annular destruction, large periannular abscesses, root pseudoaneurysms, fistula formation, PVE with root involvement, or recurrent infection after prior valve surgery [4,16,21,22].

Homografts are the preferred choice for many surgeons treating ARA because homografts adapt well to irregular surfaces, restore left ventricular outflow tract anatomy, provide resistance to reinfection, and offer excellent hemodynamic performance [61,65,66]. Moreover, homografts are suitable for repairing associated lesions of the anterior mitral leaflet [67]. Homografts are also associated with relatively low surgical mortality and reinfection rates, but concerns remain regarding availability and long-term structural degeneration. Results from the Cleveland Clinic indicate reinfection rates of 1.9% at 1 year, 7.6% at 10 years, and 14% at 20 years, suggesting a relatively low long-term reinfection risk [67].

Another option for ARR is the Freestyle prosthesis, which has a lower calcification rate and, because the Freestyle prosthesis is stentless, may allow discontinuation of anticoagulation in patients at risk of bleeding [23]. However, in patients <60 years of age with endocarditis, the Freestyle prosthesis is associated with progressive degeneration and an increased risk of reoperation [68]. Many studies suggest that the specific implant type is less important than meticulous debridement and sound reconstruction of the abscess cavity and pericardial structures [18,23,69].

During ARR, coronary reimplantation, particularly in PVE cases, is a major concern. Preoperative imaging, such as CT, is valuable for assessing coronary anatomy and abscess cavity location to minimize the risk of injury. Surgeons evaluate the direction and distance of the coronary trunks from the abscess using probes. Additionally, debridement of infected tissue near the coronary ostia can damage the ostial buttons or compromise surrounding healthy tissue. Repairing these defects with a Gore-Tex patch can enhance durability and reduce the risk of bovine pericardial calcification. Proper positioning of the left coronary ostium is vital for maintaining blood flow and preventing distortion or excessive tension, which can lead to anastomotic rupture and bleeding. In cases of severe coronary injury, coronary artery bypass grafting may be necessary, and using a slightly larger aortic root implant can help reduce anastomotic tension at the coronary sites [70].

Some authors believe that, although ARR is often performed in a higher-risk population, the lower reoperation rate may be attributable to the radical nature of the procedure [16]. Lowering the ARR threshold may reduce reoperation rates in ARA patients, including those with less severe aortic root damage (limited annular and perivalvular involvement), without increasing mortality, by providing more definitive eradication of infection.

#### 5.2.4 Double-Valve Endocarditis

Double-valve endocarditis (DVE) is more common in NVE and is associated with higher mortality and poorer long-term survival. In the German Heart Center, among 53 patients with DVE complicated by ARA, the mortality rate was 29% [71]. Similarly, a report from the University of Toronto found that among 90 DVE patients, 51% had ARA, with an in-hospital mortality rate of 15.6% [72]. Certain conditions, such as IE, degenerative calcification, and multiple prior cardiac valve surgeries, can damage the intervalvular fibrosa (IVF) between the aortic and mitral valves, necessitating IVF reconstruction, which is most commonly required in DVE with abscess formation. Major surgical approaches include the Commando procedure [73], which involves combined aortic and mitral valve replacement with left ventricular outflow tract (LVOT) reconstruction, and the hemi-Commando procedure [74], which includes AVR and reconstruction of the IVF and anterior mitral leaflet in patients whose posterior leaflet is unaffected, preserving at least the free edge of the anterior leaflet.

A study by Tomšič et al. [23] found that most infections were localized to the base of the anterior mitral leaflet, with the free edges of both the anterior and posterior leaflets remaining uninfected, supporting the feasibility of mitral valve repair (MVP). At least the intact free edge of the anterior leaflet should be preserved to provide sufficient native tissue for patch closure and pericardial patch reconstruction of the IVF. ARA involving the IVF or extending toward the anterior mitral leaflet often indicates the need for hemi-Commando surgery, in which a homograft leaflet, pericardial patch, or Dacron patch can be used [75]. Extensive damage to the anterior leaflet, the subvalvular structures, or the free edges of the posterior leaflet favors a full Commando procedure [75].

During MVP, some authors recommend using larger patches to reconstruct the anterior leaflet, thereby preventing patch retraction and postoperative stiffening. It is crucial to ensure annular stability and sufficient leaflet height to maintain long-term durability [23]. Currently, the MVP is favored over mitral valve replacement (MVR) in complex IE [76,77], as it avoids the risks of long-term anticoagulation and late prosthetic valve infection [77]. The timing of surgery is crucial; early MVP can help prevent further valve damage, but the feasibility of the approach depends on the experience and technical expertise of the surgeon [76,77].

In the study by Navia et al. [78] of 138 patients with DVE, 78% had ARA; 86 underwent the Commando procedure, and 52 underwent a hemi-Commando procedure. The Commando group had a higher in-hospital mortality rate (24% vs. 13%; *p* = 0.12). It demonstrated inferior reoperation-free survival at 1 and 5 years (83% vs. 93%; 63 vs. 93%), while the 10-year reoperation-free survival was slightly higher (50% vs. 46%). Overall, Commando surgery has been associated with higher in-hospital mortality [55,72], while hemi-Commando surgery appears to be associated with lower in-hospital mortality and better long-term survival [79]. However, when the posterior mitral annulus is involved, the Commando procedure may be more appropriate. Further prospective studies with larger sample sizes are needed to refine patient selection and procedural strategies.

## 6. Challenges and Future Directions

Surgical approaches to ARA have shown promising short- and long-term outcomes. Although current guidelines provide detailed recommendations for the diagnosis and surgical management of IE, ARA remains a life-threatening complication that requires early recognition and timely surgical intervention. However, the optimal surgical strategy according to abscess extent and anatomical location has not been clearly defined. While radical debridement remains the cornerstone of treatment, the choice of procedure is largely based on intraoperative findings and surgical judgment. Future prospective and multicenter studies are needed to clarify procedure-specific indications and improve individualized decision-making for patients with ARA.

## 7. Conclusions

For cardiac surgeons, ARA is a formidable disease because of its challenging diagnosis and complex surgical management. In this article, we summarize all aspects of this condition, aiming to provide clinical experience to help surgeons manage such cases. In conclusion, a definitive preoperative diagnosis and comprehensive evaluation based on imaging are essential for patients with ARA. Complete debridement followed by reconstruction of the affected intracardiac structures, combined with appropriate perioperative antibiotic therapy, can effectively reduce postoperative infection recurrence and optimize clinical outcomes in these patients.
