# Supplementary material for: Current Surgical Approaches to Managing Infective Endocarditis With Aortic Root Abscess
Source: Rev Cardiovasc Med. 2026 Aug 20;27(8):47800. doi: 10.31083/RCM47800 (PMC13539999; doi:10.31083/RCM47800)
Supplement: Supplementary file 1 [file 2153-8174-27-8-47800-s1.zip › Supplementary Material.pdf]

PRISMA 2020 flow diagram for new systematic reviews which included searches of databases and registers only

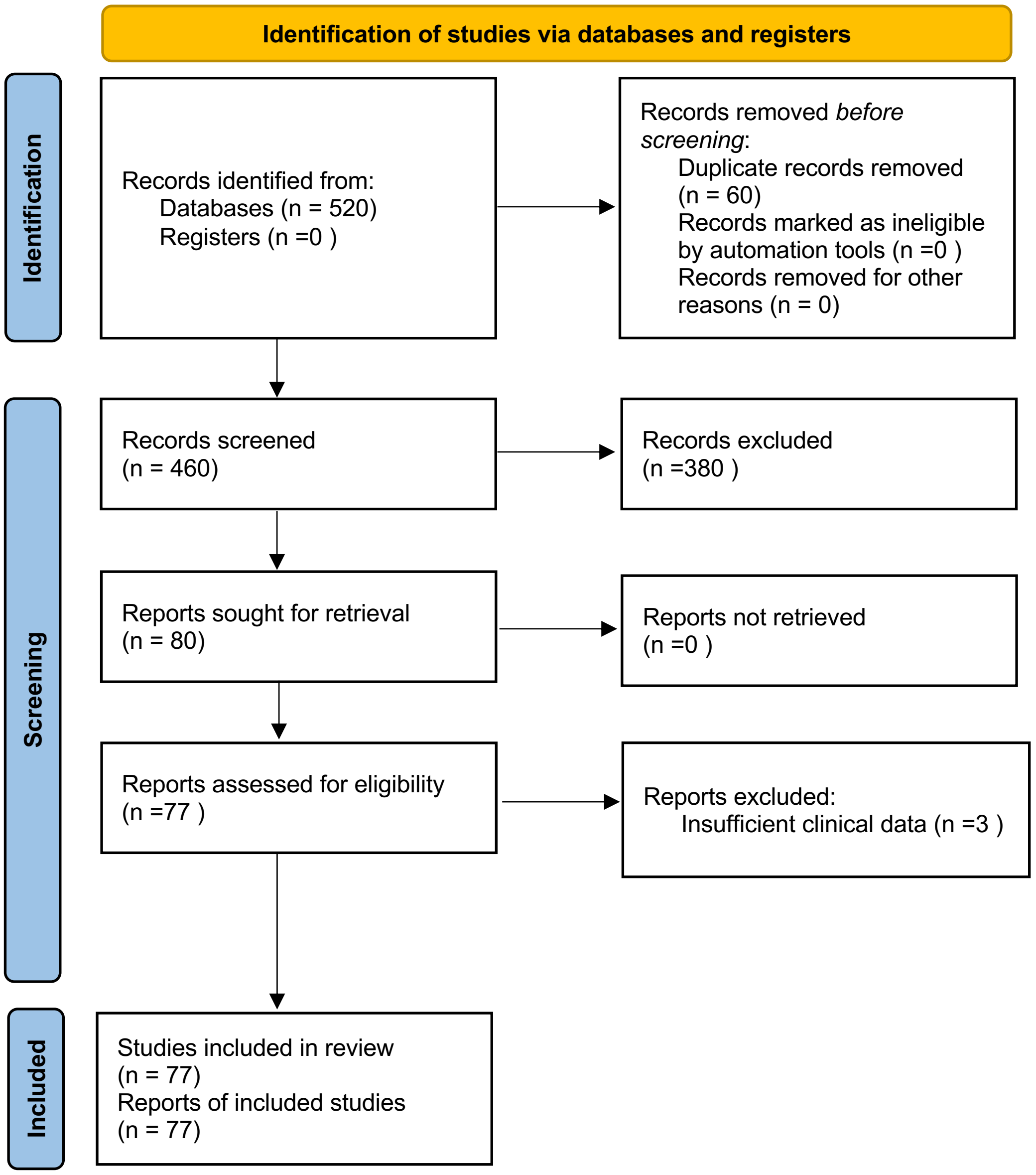

Source: Page MJ, et al. BMJ 2021;372:n71. doi: 10.1136/bmj.n71.

This work is licensed under CC BY 4.0. To view a copy of this license, visit https://creativecommons.org/licenses/by/4.0/
